# Injured bodies, damaged lives: experiences and narratives of Kenyan women with obstetric fistula and Female Genital Mutilation/Cutting

**DOI:** 10.1186/s12978-017-0300-y

**Published:** 2017-03-14

**Authors:** Lillian Mwanri, Glory Joy Gatwiri

**Affiliations:** 1 Discipline of Public Health, Faculty of Medicine, Nursing and Health Sciences, Flinders University, Level 2, Health Sciences Building, Registry Road, Bedford Park, South Australia 5042 Australia; 2 School of Arts & Social Sciences, Southern Cross University, Gold Coast Campus, Gold Coast, Australia

**Keywords:** Female Genital Mutilation/Cutting, Prolonged obstructed labour, Obstetric fistulas, Women, Kenya

## Abstract

**Background:**

It is well acknowledged that Female Genital Mutilation/Cutting (FGM/C/C) leads to medical, psychological and sociocultural sequels. Over 200 million cases of FGM/C exist globally, and in Kenya alone, a total of 12,418,000 (28%) of women have undergone FGM/C, making the practice not only a significant national, but also a global health catastrophe. FGM/C is rooted in patriarchal and traditional cultures as a communal experience signifying a transition from girlhood to womanhood. The conversations surrounding FGM/C have been complicated by the involvement of women themselves in perpetuating the practice.

**Methods:**

A qualitative inquiry employing face-to-face, one-on-one, in-depth semi-structured interviews was used in a study that included 30 women living with obstetric fistulas in Kenya. Using the Social Network Framework and a feminist analysis we present stories of Kenyan women who had developed obstetric fistulas following prolonged and obstructed childbirth.

**Results:**

Of the 30 participants, three women reported that health care workers informed them that FGM/C was one of the contributing factors to their prolonged and obstructed childbirth. They reported serious obstetric complications including: the development of obstetric fistulas, lowered libido, poor quality of life and maternal and child health outcomes, including death. Fistula and subsequent loss of bodily functionalities such as uncontrollable leakage of body wastes, was reported by the women to result in rejection by spouses, families, friends and communities. Rejection further led to depression, loss of work, increased sense of apathy, lowered self-esteem and image, as well as loss of identity and communal sociocultural cohesion.

**Conclusion:**

FGM/C is practised in traditional, patriarchal communities across Africa. Although the practice aims to bind community members and to celebrate a rite of passage; it may lead to harmful health and social consequences. Some women with fistula report their fistula was caused by FGM/C. Concerted efforts which embrace feminist understandings of society, as well as multi-sectoral, multidisciplinary and community development approaches need to be employed to address FGM/C, and to possibly reduce cases of obstetric fistulas in Kenya and beyond. Both government and non-government organisations need to be involved in making legislative, gender sensitive policies that protect women from FGM/C. In addition, the policy makers need to be in the front line to improve the lives of women who endured the consequences of FGM/C.

## Plain English summary

This qualitative research was conducted among women living with obstetric fistulas in Kenya. A fistula is an abnormal hole between the vagina and the bladder and/or rectum, developed during prolonged childbirth. The three women whose stories are presented in this paper had had Female Genital Mutilation/Cutting (FGM/C), a practice where part or all of the external genitalia is removed for non-medical reasons. Although FGM/C is widely practised and aims to bring together the community members and to celebrate girls’ passage rites into the community, it may also harm individual women, families and communities. The women describe how their lives were negatively affected by FGM/C and fistulas. The occurrence of fistula and subsequent leaking of body wastes, resulted in rejection of the women by their spouses, families, friends and communities. This rejection further led to mental, physical and social suffering. We found that the women perceived that FGM/C was the cause of the prolonged and obstructed labour that led to the fistula, and we concluded that, among this small sample, FGM/C practices were harmful to women both physically and socially. We recommend concerted multi-sectoral, multidisciplinary and community development responses to address issues of FGM/C in communities. These activities may reduce the prolonged labour cases that may lead to obstetric fistulas in Kenya and beyond.

## Background

Female Genital Mutilation/Cutting (FGM/C) or female circumcision is a mutilating procedure which is practised in many regions across the world among ethnic and sociocultural groups, and across religious beliefs such as Islam and Christianity [1, 2]. FGM/C is defined as, ‘a practice that removes partial or total parts of external female genitalia or causes other injury to the female genitalia for non-medical reasons’ [3, 4]. Four main types of FGM/C have been described including: (i) type 1 which involves partial or total removal of the clitoris and/or the prepuce (clitoridectomy), (ii) type 2 which involves partial or total removal of the clitoris and the labia minora, with or without excision of the labia majora, (iii) type three or infibulation, which is the most serious and invasive type and involves narrowing of the vaginal orifice with creation of a covering seal by cutting and appositioning the labia minora and/or the labia majora, with or without excision of the clitoris, and (iv) type four which involves all other harmful procedures to the female genitalia for non-medical purposes, for example: pricking, piercing, incising, scraping and cauterization [2–5].

The procedure is practised in more than 28 African countries, as well as some countries in Middle East, South America and Asia, with prepubescent girls being the main target population [4]. Global estimates account for more than 200 million FGM/C cases, with Africa including Kenya contributing more than 91.5 million cases [4, 6, 7]. It has also been reported that migrants from countries where FGM/C is traditionally practised, may continue the practice when they migrate to higher resourced countries, such as United Kingdom, Australia, Switzerland, Canada, the United States, France and Sweden [8–10].

In traditional African patriarchal communities, social norms dictate communal activities and events such as performing FGM/C especially on young women as part of initiation rituals into adulthood [11–13]. Although these practices are meant to bring community members together, including celebrating the passage rites of girls to women, they may be associated with harmful health, psychological and social consequences on individuals, families and communities [14]. The impacts of FGM/C include short term and long term health complications leading to physical, psychological and socio-cultural problems among affected individuals. Short term health complications include, but are not limited to bleeding, pain and shock [3, 4], while chronic pain, genitourinary tract infections, damage to genitalia, postpartum haemorrhage, genital tissue scars and keloids, anaemia, and in severe cases, maternal and foetal deaths being the known long term complications [3, 5, 15, 16]. In addition, FGM/C has been associated with serious psychological problems, such as anxiety, post-traumatic stress disorders and psycho-sexual conditions leading to bodily identity problems [6, 7]. Some women who have experienced FGM/C have prolonged and/or obstructed labour, which may lead to the development of obstetric fistula(s) [4]. A vaginal obstetric fistula occurs when a hole (fistula) forms between either the vagina and rectum (rectovaginal fistulas-RVF) or between the vagina and bladder (vesicovaginal fistula -VVF) following prolonged childbirth complications [17]. Among other complications, a woman with a vaginal obstetric fistula may develop urinary and/or faecal incontinence, leading to severe physical, psychological and socio-cultural problems for the women, their families and the entire affected communities [18]. Fistulas cause complications such as foul smelling, vaginal and/or rectal discharges, urinary tract infections, dyspareunia and uncontrollable flatulence [7].

There is some evidence of an association between FGM/C and obstructed/prolonged labour, and a large body of evidence exists for a causal relationship between prolonged/obstructed labour and obstetric fistula [4, 19, 20]. However, The World Health Organization only presumes that both conditions could be linked in women living with both FGM/C and fistulas, but does not confirm a direct or causal relationship between FGM/C and fistulas.

As already established in existing literature, FGM/C practice is well rooted and grounded in patriarchal cultural and traditional beliefs, but the ideological drivers of FGM/C vary significantly in Kenya by location and ethnicity [6]. On one hand, the traditional collectivist patriarchal communities practise FGM/C as a way to bring communities together and to celebrate the initiation of young women to adulthood [11]. On the other hand, the same collectivist and patriarchal societies tend to marginalise women and girls, and as such, women have less power compared to men, who make most decisions, including what can be done to women’s body (e.g. FGM/C) [14, 21]. The FGM/C practice is usually performed by older women who believe that the procedure will increase women’s attractiveness and marriageability [11]. The practising women are themselves products of a patriarchal society who perform FGM/C on younger girls to achieve a cultural standard expected for women in such communities [5, 11, 17]. For many women who have had FGM/C, their traumatic experiences are not discussed and the silence surrounding the traumatic experiences complicates any eradication efforts [14]. Addressing this and other culturally fuelled social problems requires a deep understanding of the local contexts, including the sociocultural psychosexual, religious and economic factors that perpetuate such practices [14]. Similarly, the voices of affected women need to be the centre of the discourse and to be used in developing FGM/C preventative strategies.

This paper focuses on stories of three women who reported developing fistulas post FGM/C. The aim is to provide rich insight and perspectives of women’s experiences of living with fistulas linked with FGM/C in Kenya. The paper presents only part of findings from a larger dataset collected for the doctoral program of the second author (GG). The aim of the larger study was to explore the experiences of women living with fistulas in Kenya, experiences that are hidden, inaccessible, suppressed and ignored.

## Methodology

### Theoretical frameworks

The study was guided by theoretical frameworks including the Social Network [22], and the African Feminist Theories [23, 24]. The Social Network Theory (SNT) ‘analyses communities and/or settings to depict patterns of factors and dynamics in a society, common traits, composition of the community and the social structures that orchestrate community practices and actions [22]. We have used SNT in recognition of the African (including Kenyan) collectivist characteristics/behaviours, where the idea of the community as a collectivist is commonly accepted to influencing actions in the entire and/or part of the community [25]. The African communal way of life and social networking, brings together family members, relatives and other people forming ‘a community,’ that is heavily involved in communal events [12], which may include – as would be expected in the current study – the celebration of the FGM/C practices which are performed to initiate young women to adulthood. These traditional community networks and dynamics are very complex but important, as they provide communities with identity, a medium for shared experiences and a sense of belonging [26]. However, the same traditional networks and dynamics place superiority on men and expect obedience and submissive behaviours from women, inadvertently causing harm when some actions dictated by men cannot be questioned by women [14, 27]. In the case of FGM/C, a group of ‘older women or elders’ become instrumental in encouraging the FGM/C practice on ‘younger women’ [4, 6, 28]. In addition, the overarching patriarchal constructions encourage women to be actors of mutilating their own bodies through FGM/C in order to achieve a higher socially acceptable status in the community [21]. In seeking to fit into the community, women ‘sacrifice’ the wholeness of their bodies only to suffer consequences of multiple losses, including loss of social networks and community support if they develop obstetric fistulas associated with FGM/C [11].

The other approach that we have used to analyse the FGM/C cases more intimately is the Feminist Theory (FT) [23]. We have applied this theory to support, among other arguments, the notion that FGM/C is used to control women’s bodies and sexuality [24]. There are traditional beliefs in some cultures about women’s bodies that orchestrate negative feelings of insecurity and are intermingled with discourses about ‘how to get and keep a man’ [14, 29]. Beliefs such as the clitoris embodying a masculine characteristic makes men fearful of engaging in sexual intercourse with ‘a fellow man’ which encourages women to ‘fix this anomaly’ with genital cuttings from ‘village midwives’ [30]. These traditional patriarchal notions prey on women’s insecurities about their body images and are ably assisted by discourses about ‘what makes a woman complete.’ Girls are socialised to believe that being uncircumcised is unattractive to men rendering them ‘incomplete’, hence unmarriageable [31]. In the cultures where FGM/C is prevalent, a ‘normal’ and celebrated body is one that has been cut/mutilated for the purposes of ‘disciplining’ it. As Foucault’s theory suggests [32], the construction of a ‘normal’ body interacts with traditional and patriarchal notions in order to produce docile and disciplined bodies that are easy to control. FGM/C also renders women’s bodies as sites of oppression by way of sexualization and modification in order to meet the patriarchal desires [11]. The sexualization and the grooming of girls bodies, for the benefit of men, begins at an early age [33]. This can limit educational achievements, underpin gender inequality, and serve to perpetuate associations of masculinity with predatory sexual prowess and justify sexual violence and silencing of women’s needs. FGM/C may not only contribute to women’s risk to develop fistulas, but may also limit their ability to seek or access medical support once they develop fistulas [11, 19, 34].

### The FGM/C practice in the study setting

Kenya is an East African country where FGM/C is still heavily practiced [31]. In the context of traditional African patriarchal communities, the practice of FGM/C is perpetuated by community traditions and norms and is enforced by older women who need to conform to these norms, and hence feel obliged to conduct FGM/C on themselves and others [13]. The prevalence of FGM/C varies according to ethnic groups and geographical settings, with the North-Eastern and South-Western part of the nation being over represented [6]. This variation in practice is influenced by factors including socioeconomic status, overall education levels of local people and cultural beliefs, with the prevalence reported to be lower in urban areas where cultural ties and beliefs are not staunchly observed. The overall prevalence of FGM/C among Kenyan women population is reported to be 28%, a total of 12,418,000 women [6], indicating the need to address FGM/C as a significant public health issue. In this study, participants were from various regions in Kenya and were admitted for surgical fistula repair at the Kenyatta National Hospital in Nairobi–the capital city of Kenya, and in the Gynocare Care Clinic in Eldoret. Participants were from different tribes, and most of them lived in rural and remote settings where obstetric and medical services are barely accessible if at all they exist.

## Methods

### Study design and data collection

In order to explore the context underpinning FGM/C practices and to generate rich information from participants, a qualitative research method was employed [35]. The study participants were women waiting to undergo surgical fistula repair at Kenyatta National Hospital in Nairobi and the Gynocare centre in Eldoret, Kenya. Participants were recruited through a purposive method because the study was centred on a specific research objective. An introduction letter describing the study and including the researcher’s telephone number was sent to the head nurses in the fistula wards at the two hospitals to seek permission to undertake the research. The researcher was invited to introduce herself and explain the research aims to the medical team in the fistula care wards. The nurses in the fistula clinics then passed the information to all the fistula patients seeking treatment. Those women who expressed interest in hearing more, joined the researcher in a private designated room (which had been booked previously) so that more information about the research could be provided on a one-to–one basis. All participants were screened according to the eligibility criteria before they were invited to participate. To participate, one had to be a Black Kenyan woman who had the experience of living with vesico- and/or recto-vaginal fistula. One had to be in a treatment facility seeking treatment for fistula, be over 18 years and fluent in Swahili (The national language in Kenya). After passing the criteria, the researcher explained the informed consent in Swahili and ensured that participants understood the following:

1. Why they were participating in research,
2. The purpose, the procedures and the ethics of the research
3. The risk and benefits of the research
4. The voluntary nature of research participation and their right to stop at any time they wished without any consequences to them.
5. The procedures used to protect confidentiality.

After communicating the above information effectively, participants were offered an opportunity to ask questions or raise any concerns before being invited to sign the consent form before commencement of the interviews.

Face to face, semi structured, open-ended interviews lasting approximately one hour were conducted. The researcher who collected the data was a Black Kenyan African woman with firsthand knowledge on the cultural practices and spoke fluent Swahili. This was important as the advantages of being an ‘insider’ researcher helped to create rapport quickly and to communicate effectively. Interviews were conducted privately inside a private room in the treatment facilities or outside in a quite garden as preferred by some of the participants. Asking open ended questions such as ‘what do you make of your experience?’ or ‘tell me about your experience’ gave them an opportunity to give subjective and personal narrative of what living with vaginal fistula meant to them. The information about FGM/C was self-reported by one participant at the beginning of the interviews when asked to explain what they thought the cause of their fistula was. In subsequent interviews, if the woman did not spontaneously mention FGM/C, the researcher probed if the women had FGM/C. For the purposes of this paper, only the narratives of the women who made the connection between their FGM/C and fistulas are included, as the authors wanted to demonstrate the relationship of the two. All the interviews were audio-recorded and data files password protected.

### Data analysis

After the translation (by the second author and verification by the first author) into English, interview transcripts were transcribed by the second author. Both authors speak Swahili and English fluently, making it easy to verify for accuracy and the quality of translation. The full 30 transcripts were repeatedly read by both authors and data were analysed using a framework analysis as suggested by Ritchie and Spencer [36]. However, this paper descriptively analyses narratives of three women who linked FGM/C and their fistulas. Their stories were powerful and informative, compelling the authors to write a specific paper about them and to enrich the body of knowledge about negative health/social impact of FGM/C on women who undergo the procedure. The paper presents the stories of three women as a case study.

## Results

### Characteristics of participants

A total of 30 women aged between 18 and 68 years were interviewed. These women had lived with vaginal fistulas for periods ranging between 11 months to 40 years. Most of the participants developed fistulas due to prolonged and obstructed labour and had lost all or some of their babies.

Out of the 30 participants who were interviewed, three women reported that they believed the FGM/C contributed to their fistula. They all also reported that health care workers had told them at the time they experienced the fistula that their FGM/C was the “cause” of their fistula. Twenty-one participants said they were never circumcised and six women reported that they did not see any correlation between FGM/C and their fistulas. These six women reported they either did not know if there was a correlation between FGM and fistula or they reported that FGM/C was a ‘normal’ practice that ‘every’ woman goes through so it must have been them and not the FGM/C procedure that was problematic.

The profile and characteristics of the study participants are presented in Table 1.

**Table 1:** Sociodemographic characteristics of participants

Participant	Age	Fistula type	Cause	FGM/C	Related FGM/C to fistulas	Years with fistula	No of children	Income/M	Educational level	Marital Status
1.	58	VVF	OPL	Yes	No	22 years	11 (3 dead)	None	None	Widowed
2.	48	VVF	Chemo	No	NA	3 years	4	< average	college	Married
3.	39	VVF	PL	No	N/A	1 year	5	< average	None	Married
4.	42	RVF	OL	No	N/A	6 years	3	average	college	Married
5.	38	VVF	OPL	No	N/A	1 year	1	< average	Primary school	Separated
6	18	VVF	PL	Yes	No	1 year	None	None	Class 7	Single
7.	35	RVF	PL	No	N/A	7 years	7	< average	Primary school	married
8	26	RVF	OL	No	N/A	I year	1	< average	Sec School	Married
9	22	VVF	PL	No	N/A	2 Years	None	None	None	separated
10	22	VVF	PL	No	N/A	1 year	1	< average	None	married
11	28	VVF	OPL	Yes	No	13 years	None	None	Class 7	separated
12	50	VVF	PL	No	N/A	22 years	6	None	None	widower
13	40	VVF& RVF	OPL	Yes	Yes	22 years	5	< average	None	married
14	61	VVF	OPL	Yes	No	40 years	2 (both dead)	None	None	Divorced
15	45	VVF	OL	Yes	Yes	21 years	1 (1 died)	None	None	Separated
16	20	VVF	OL	No	N/A	I year	1	None	College	separated
17	22	VVF & RVF	OPL	Yes	Yes	10 years	1 (1 dead)	None	None	separated
18	30	RVF	No sure	No	N/A	20 years	1	< average	college	Married
19	18	VVF	Rape	No	NA	11 years	None	None	Class 7	single
20	32	VVF	PL	Yes	No	1 year	2	None	None	Divorced
21	45	VVF	Negligence	No	NA	4 years	7	< average	Primary school	married
22	22	VVF	OPL	No	N/A	7 years	None	None	Some college	separated
23	29	VVF	OPL	No	N/A	18 years	None	None	Some primary school	Divorced
24	28	VVF	OL	No	N/A	1 year	6	< average	Primary school	married
25	30	VVF	OL	No	N/A	8 years	None	< average	Sec School	married
26	43	VVF	OPL	Yes	No	1 year	6 (2 dead)	< average	Primary school	married
27	40	VVF	OL	No	N/A	-	5	< average	Primary school	married
28	22	VVF	OL	No	N/A	2 years	None	None	Sec School	Single
29	21	VVF	PL	No	N/A	1 year	None	None	Sec School	Single
30	Unknown	VVF	PL	No	N/A	Unknown	8 (all dead)	None	None	Widowed

*OPL* obstructed prolonged labour, *PL* Prolonged labour, *OL* Obstructed labour, *RVF* Rectal vaginal fistulas, *VVF* Vesical vaginal fistulas, *N/A* Not applicable

### Stories women in the case study

Stories in this paper include narratives from three women “Moraa”, “Sasha”, and “Chemutai” (all pseudonyms), who had undergone severe FGM/C (infibulation) that were described to them as the cause of their prolonged/obstructed labour which led to the development of vaginal obstetric fistulas. The women came from three tribes in Kenya: Kisii, Samburu and Kalenjin. Their narratives are powerfully described, and include heart breaking recounting of two painful events in their lives-the day they were circumcised and the day(s) of childbirth.

#### Sasha’s story

Sasha is a 22-year-old woman; she is currently separated from her husband who married a second wife shortly after she developed the fistula. Sasha was first pregnant when she was 11 years and after a traumatic birth and labour which lasted 6 days, she was finally able to birth a dead, macerated baby. She collapsed after this ordeal, but soon after she woke up, she discovered a pungent smell and realised that her body was now incontinent of both urine and faeces. Sasha hails from one of the remotest parts of Kenya in Samburu-a community that has over 90% FGM/C rates. She was married at 9 years of age shortly after the FGM/C ceremony, a ritual that is considered a rite of passage to adulthood in the Samburu culture. Sasha reported that doctors at the hospital informed her that her severe infibulation contributed to her difficult childbirth.

*‘You know in Samburu, it is expected that you will do this thing* [FGM/C]. *They took me early in the morning and poured really cold water on me. It was so painful but I was not allowed to scream… when I developed this problem* [fistula] *and came to this hospital, one of the nurses said that this thing* [FGM/C] *and the way it was done, had contributed to me having this urine problem’.* (Sasha, 22 years old)

Women can develop vaginal fistula following infibulation, which is the most severe and disfiguring form of FGM/C [5]. As with Sasha, her young body was wounded by the injuries of FGM/C forming a tough scar that was difficult to open during coitus and childbirth. This is in addition to the fact that Sasha was not sufficiently developed to deliver a child at 11 years. Without access to Emergency Obstetric Care, her baby was stuck in the birthing canal, causing vaginal tissue necrosis [37]. FGM/C along with child marriages provides a double tragedy for young girls like Sasha.

In addition to the physical injury and pain, women who are in situations such as Sasha’s, suffer isolation and ridicules not from strangers, but their own families and communities. In such situations, women’s psychological mental health status is affected, the condition that may trigger suicidal ideation. These assertions can be depicted in such statements as below:

‘….*. Especially when the urine passes it burns you so much you turn completely red. So every time I was sick people would say that I am lying or that am pretending. When you say or do something they tell you to go away with your urine or your faeces. My husband would tell me that I would forever leak urine that it would never go away. It would make feel like I wanted to die…..’* (Sasha, 22 years old)

#### Moraa’s story

Moraa is a 40-year-old woman who developed a vesico-vaginal fistula when she was 18 years following a complicated, prolonged, 2-day labour. She has been married for 23 years and has five surviving children. She hails from Kisii, a heavily patriarchal tribe in Kenya and gives insight to one of the health implications of FGM/C. She says:

*‘When they cut me, my body was never the same again. I really struggled to push the baby that gave me this problem* [fistula]. *It almost killed me… also it’s impossible to feel anything* [meaning sexual pleasure] *when circumcised…That is why Kisii men are not marrying fellow Kisii women because they say that we are like stones. They want to go to women who get turned on just by a simple touch’* (Moraa, 40 years old).

Research has demonstrated that FGM/C may negatively affect women’s libido and sexual sensations [21, 30, 38]. When the clitoris, which is the most sensitive part of the woman’s body and is purposely meant for sexual pleasure, is mutilated, women find it harder to attain orgasm or experience sexual pleasure. This achieves one of the principal patriarchal reasons for FGM/C, which is to control women’s bodies and produce bodies that are submissive and docile sexually. Moraa continues by warning women that FGM/C is a community expectation.

*‘The doctor said that if women can delay getting children and not get cut* [FGM/C] *this kind of problem can be avoided. The doctors said that if I had waited having children and not been cut, I would not have the problems I have now. I was very young when they did it* [FGM/C] *to me and back home most girls must do it. If you don’t do it, people laugh at you and you will not find someone to marry you’* (Moraa, 40 years old).

In the Kenyan Kisii community for instance, FGM/C can be understood as a means to modify a woman’s body in order to make it more desirable to a potential male mate. Literature on FGM/C gives some further insights into the need for a conforming body and African women’s participation in numerous patriarchal expectations is mostly in preparation for their betrothal and social acceptance [29, 39–41]. Even after women are married, keeping their bodies primed and acceptable for their husbands is a lifelong endeavour. The aspect of choice is removed from the young women, who may not understand the potential lifelong consequences of FGM/C such as vaginal fistulas, pain, infection, and distorted body identities.

#### Chemutai’s story

Chemutai, who is a 45 years old woman, lives in Chepkoilel in Uasin Gishu County and narrated her own experience of FGM/C and fistula. Similar to stories of the other women in this paper, Chemutai reported her belief that her birthing complications resulted from severe FGM/C. She reports that she could not stand or walk without support when the procedure was performed and that it left her with permanent damage that complicated her marriage and the delivery of her two children who were born dead (still birth).

*‘I will never forget that night. It was cold and they held me down and did what they did. First they poured really cold milk on me. They said that would help with the pain. It took me a long time to heal. Going to ease myself was so hard… I did not know the way they cut* [FGM/C] *me had anything to do with this urine problem, but when I came here* [Gynocare clinic, Eldoret] *the nurse told me that I had a big scar and that it might have prevented my children from coming out properly. I am childless now and my husband left me when he saw that this problem was not going away’ (*Chemutai, 45 years old).

Although FGM/C is widely celebrated and is a belief that it can enhance marriageability, it is reasonable to state that potential serious complications such as fistulas are not well understood by the wide FGM/C practising community. As such, when women who have undergone this procedure develop such complications, they shun away from the same communities for fear of further harm arising from the lack of understanding and lack of empathy from the people surrounding them. These arguments can be illustrated by statements such as:

*‘….the reason why I don’t tell people I have this condition* [fistula] *is because most of them will think I am cursed, because not many know about this illness’* (Chemutai, 45 years old)

## Discussion

Drawing from both the current study findings and the literature, it is plausible to reiterate that FGM/C is a significant public health problem in Kenya, and globally [2, 42]. Severe infibulation may be associated with serious sociocultural, economic and health complications such as obstetric fistulas [43], as demonstrated by the women’s stories in the current study.

In addition to direct complications as stated above, evidence exists implicating FGM/C as one of the risk factors for HIV transmission in communal settings, as it involves the sharing of cutting instruments such as knives and razors [12, 22, 44].

Consistent with the Afro-communitarian ethos, large groups of girls are brought together from networks of close knit communities to undergo FGM/C as part of socio-cultural initiation rite and celebrations [13]. However, the narratives from Moraa, Sasha and Chemutai, inform of the rejection and isolation suffered by women following the development of vaginal fistulas that they believed were due to the FGM/C. Though FGM/C is a practice that is perceived as bringing the community cohesion, developing complications from the practice might render some women as social pariahs. This rejection and isolation of sick community members is counter to the Afro- communitarian ethos, which embraces communalism. Because of their circumstances such as leaking dirty wastes and bad body odours, these women, not only endure the biomedical complications of fistulas, but also undergo loss of multiple statuses including: the loss of loved ones (e.g. husbands, children, sibling or even parents), extended families, the community and broader social networks. These losses further complicate women’s physical and mental health leading to a further vicious cycle of poor health, poor socioeconomic outcomes and further disadvantages [45].

Feminist theories would also suggest that the difficulties experienced by the women, contribute in the oppressive structuring that keeps them subsumed and subordinated not only by patriarchy, but by their own difficult existence [11, 27]. Similarly, traditions such as FGM/C continue to subsume women into an oppressive thinking that enables their own subjugation. The traditional structures operate broadly and reinforce negative attitudes towards women that lead to gendered oppression. As Salami posits [46], the biggest challenge that African feminists have is to challenge cultural traditions that oppress women, such as FGM/C. This may mean reclaiming the power that women inherently deserve from men. Reclaiming power is necessary because as it is typical in any patriarchal society, men tend to horde most of the social economic and political power, while women are expected to be submissive and subservient [47]. As described in the stories of Moraa, Sasha and Chemutai, the value of women with fistula is far below that of other women particularly due to their incontinence. With lower literacy, lower or no employability, most have little or no option but to accept their ‘failure’ and status quo, instead of challenging the customs and expectations of their communities, in which they must live regardless of the harms those communities may have caused them. However, as much as the discourses surrounding the debates on FGM/C are necessary, they need to move beyond academic theorisations to an engagement with the women with FGM/C and fistulas.

The FGM/C practice and fistulas not only have significant impact on the individual, families and communities, they also place a significant burden on health systems and should be a matter of public health policy [2, 31, 34, 42]. FGM/C and fistulas, often lead to patients needing caesarean sections (for those who can access emergency obstetric services), extended hospital stay, and in some instances, the loss of life for mother and/or child [4, 34]. This comes with significant cost to both the community and health care system which loses productive members of the community to preventable conditions and the cost repairing the fistulas [9].

Although globally there have been multitudes of efforts to combat FGM/C [2, 42], addressing FGM/C remains challenging due to its long historical and traditional practice and acceptance. Respected ‘older women’, who are elders/leaders in their communities, and were themselves ‘*victims*’ of FGM/C, advocate for FGM/C to be performed on their children and younger women in their communities [4, 31]. Older women often are in the frontline performing FGM/C due to cultural communitarian ethos and patriarchal system that, as stated elsewhere in this paper, stratify women in order of importance and status depending on whether they have had FGM/C or not. In addition, in some community settings where FGM/C is known to be harmful, efforts have been made especially by ‘caring’ parents and significant others to make it *“safer,”* through seeking help from medical professionals, through what is known as ‘*Medicalization of FGM/C’* [7, 35, 48]. The medicalization of FGM/C has also been a hindrance to the success of public policy and other initiatives against the practice [17, 18]. Even though it is now common knowledge that FGM/C carries no medical benefits, in some communities, its medicalization has perpetuated the practice due to the high respect afforded to medical practitioners and the false belief that it is ‘*safer’* [6, 9] when performed by clinicians.

Interestingly, those women who reported that their fistulas were related to FGM/C seemed to do so as a result of direct conversations they had with health workers *after* developing the fistula. This may imply that there is an underlying gap of information on how FGM/C can cause long term scarring of tissues that can lead to obstructed and prolonged labour. However, we did not specifically examine this issue.

Despite the existence of strong traditions of African communitarian, which provide social networks, identity and sense of belonging to communities [12], the power inequalities between women and men seem to be problematic in patriarchal communities such as in Kenya. This paper demonstrates that due to such power imbalances and marginalisation of women, FGM/C is practised without considering what harmful impacts it can cause on women and those surrounding them.

To redress these issues, there is a need for a strong advocacy and employment of multiple strategies including involving community education and social change. A complete cultural shift in the way people think about women and their bodies is necessary in order to completely eradicate FGM/C and other similarly injurious practices [34, 49]. This will involve the collaboration of political, religious and community leaders as well as the involvement of grass root community based organizations to inform alternative methods of ‘initiation’/rites of passage [31, 49]. In addition, engaging both older and younger women and improving their affirmative responses towards such socio-cultural norms underpinning these practices, would reduce the likelihood of them romanticising this harmful traditional practice that promotes gendered oppressive negative attitudes towards women [50].

Additionally, community development strategies and community based participatory approaches such as those developed by Paulo Freire, would encourage critical self-empowerment of women [51, 52]. Furthermore, Freire advocates for critical community practice where members of the community are able to identify institutionalised forms of oppression and derive ways of subverting that systemic power. The community participatory approaches will bring together people with a collective objective to empower the most marginalised persons in the community [52]. Similarly and consistent with the principles of primary healthcare and the Ottawa Charter [3, 15], inter-sectoral collaboration involving both health and non-health sectors, using both top down and bottom up approaches, will be required necessitating resource reallocation to ensure that women do not continue to endure such horrific practices in the name of culture and traditions [15]. Moreover and also importantly, efforts need to be made to educate men–especially on the importance of supporting women who refuse FGM/C. Feminist thinking would suggest that men who exist outside of the patriarchal expectations are more likely to see women as their equal rather than as their subordinate.

This study has limitations including that only a small number of participants’ narratives were presented in this paper. Although a purposive sample was used to interview women living with fistulas in Kenya and identified those who linked their fistulas with FGM/C, the results do not necessarily reflect the experience of all women who have had FGM/C or who are living with fistulas. The study also did not explore issues such as what activities health workers can do to prevent FGM/C and fistulas in their communities. These are important issues that could be explored in future studies.

## Conclusion

This paper has used the stories of Sasha, Moraa and Chemutai to discuss the relationship between FGM/C and vaginal fistula. Given that FGM/C is widely practised in Kenya, it potentially increases the rate of obstetric fistulas in the nation. FGM/C practices also pose major cultural, psychosocial and health consequences in affected populations, which may lead to negative economic and social consequences for individuals, families, communities, and the country. We believe that FGM/C is a violation of human rights and it serves no medical or cultural purpose. We also believe that vaginal fistulas might be reduced if FGM/C was eradicated. A comprehensive response employing principles of primary health care and feminist community development approaches, with the political goodwill of governments, civil societies and communities, is needed. Women who have had FGM/C or who are at risk of being cut/mutilated need to be empowered so that they can advocate against the practice. Preventing FGM/C may reduce childbirth complications and other lifelong consequences, including increases in health care cost for both the individual and the nation [7, 53]. Moreover, while the relationship between FGM/C and vaginal fistulas seems obvious, there is inadequate research to show causality. More research is needed to uncover the extent to which FGM/C is associated with obstetric fistulas and to discover new methods to prevent FGM/C.

## Abbreviations

FGM/C: Female Genital Mutilation/Cutting
FT: Feminist Theory
KES: Kenyan Shillings
KNH: Kenyatta National Hospital
RVF: Rectovaginal fistulas
STN: The Social Network Theory
US$: United State of America dollars
VVF: Vesicovaginal fistula
